# Urocortin 3 Levels Are Impaired in Overweight Humans With and Without Type 2 Diabetes and Modulated by Exercise

**DOI:** 10.3389/fendo.2019.00762

**Published:** 2019-11-06

**Authors:** Sina Kavalakatt, Abdelkrim Khadir, Dhanya Madhu, Maha Hammad, Sriraman Devarajan, Jehad Abubaker, Fahd Al-Mulla, Jaakko Tuomilehto, Ali Tiss

**Affiliations:** ^1^Research Division, Biochemistry and Molecular Biology Department, Dasman Diabetes Institute, Kuwait City, Kuwait; ^2^Research Division, Dasman Diabetes Institute, Kuwait City, Kuwait; ^3^Department of Public Health, University of Helsinki, Helsinki, Finland; ^4^Department of Public Health Solutions, National Institute for Health and Welfare, Helsinki, Finland

**Keywords:** urocortin, UCN3, CRF, obesity, diabetes, exercise

## Abstract

Urocortin3 (UCN3) regulates metabolic functions and is involved in cellular stress response. Although UCN3 is expressed in human adipose tissue, the association of UCN3 with obesity and diabetes remains unclear. This study investigated the effects of Type 2 diabetes (T2D) and increased body weight on the circulatory and subcutaneous adipose tissue (SAT) levels of UCN3 and assessed UCN3 modulation by a regular physical exercise. Normal-weight (*n* = 37) and overweight adults with and without T2D (*n* = 98 and *n* = 107, respectively) were enrolled in the study. A subset of the overweight subjects (*n* = 39 for each group) underwent a supervised 3-month exercise program combining both moderate intensity aerobic exercise and resistance training with treadmill. UCN3 levels in SAT were measured by immunofluorescence and RT-PCR. Circulatory UCN3 in plasma was assessed by ELISA and was correlated with various clinical and metabolic markers. Our data revealed that plasma UCN3 levels decreased in overweight subjects without T2D compared with normal-weight controls [median; 11.99 (0.78–86.07) and 6.27 (0.64–77.04), respectively; *p* < 0.001], whereas plasma UCN3 levels increased with concomitant T2D [median; 9.03 (0.77–104.92) *p* < 0.001]. UCN3 plasma levels were independently associated with glycemic index; fasting plasma glucose and hemoglobin A1c (*r* = 0.16 and *r* = 0.20, *p* < 0.05, respectively) and were significantly different between both overweight, with and without T2D, and normal-weight individuals (OR = 2.11 [1.84–4.11, 95% CI] and OR = 2.12 [1.59–3.10, 95% CI], *p* < 0.01, respectively). Conversely, the UCN3 patterns observed in SAT were opposite to those in circulation; UCN3 levels were significantly increased with body weight and decreased with T2D. After a 3-month supervised exercise protocol, UCN3 expression showed a significant reduction in SAT of both overweight groups (2.3 and 1.6-fold change; *p* < 0.01, respectively). In conclusion, UCN levels are differentially dysregulated in obesity in a tissue-dependent manner and can be mitigated by regular moderate physical exercise.

## Introduction

Chronic metabolic stress and low-grade inflammation are key factors in obesity, insulin resistance and Type 2 diabetes (T2D) and involve the impairment of mechanisms in both central and peripheral tissues (1). Corticotropin-releasing factor (CRF) neuropeptides are involved in such mechanisms (2). In mammals, the CRF family comprises four structurally related ligands, CRF, and urocortin (UCN) 1–3 (3, 4), which have been implicated in stress adaptation responses and energy balance regulation (2) and exhibit proinflammatory and/or anti-inflammatory effects depending on the context and disease (5, 6). CRF peptides were initially discovered in the brain but are also expressed in peripheral metabolic tissues, including skeletal muscle and the pancreas (7, 8). Although the CRF system is not yet fully understood, its modulation has been proposed as treatment for human metabolic disorders (5). The genetic manipulations of the CRF family in mouse models has demonstrated distinct metabolic phenotypes, thus suggesting different but crucial endogenous roles for each of these peptides and their receptors in metabolic pathways (9).

UCN3 is the least studied member of the CRF family, but it is known to bind and signal selectively via CRF receptor 2 (CRFR2) (10, 11). UCN3 is highly expressed in the pancreas, where it is exclusively expressed by β-cells in mouse islets but is expressed by both β-and α-cells in humans (12, 13). The abundance of UCN3 is decreased in β-cells in patients with T2D or in macaques fed with a high-fat diet (12). UCN3 enhances glucose-induced insulin secretion via endogenous and paracrine action in the pancreas, ensuring the coordinated release of insulin and glucagon (12, 14). The decreased expression of UCN3 in pancreatic β-cells in patients with diabetes revealed the loss of this feedback mechanism (12). UCN3 also contributes to energy homeostasis in key peripheral metabolic tissues (15, 16). In line with this, Global overexpression of UCN3 in mice resulted in resistance to the adverse metabolic effects of a high-fat diet while local UCN3 expression in muscle enhanced glucose disposal and signaling in this tissue (17). In a cross-species association study, UCN3 was proposed to be linked to obesity due to its location on human chromosome 10p15.1 where quantitative trait loci for obesity have been reported (18).

Increased expression of UCN3 is thought to protect from high-fat diet-induced hyperglycemia and can increase energy expenditure (9).Therefore, UCN3 represents a potential therapeutic target for metabolic diseases management. However, the distribution of CRFR2 and UCN3 in adipose tissue and the mechanisms underlying their role in obesity and adipose tissue-related insulin resistance remain unclear. Despite the pivotal role of UCN3 in energy homeostasis and insulin secretion, the circulating and adipose tissue levels of UCN3 in obese people with or without T2D have not been previously reported.

Regular physical exercise has become a key component in the non-pharmacologic treatment and lifestyle management of people with obesity and diabetes (19, 20). Several studies reported a reduction in the incidence of diabetes among physically active individuals (21). Beneficial effects of exercise go beyond body weight loss and include enhanced insulin sensitivity and mitochondrial function and lowered risk of cardiovascular diseases (22). Previous studies including ours have associated the protective effects of exercise with improved inflammation and metabolic stress responses (20, 23–25). Furthermore, exercise affects various central nervous systems related to energy homeostasis and food intake via the activation of the CRF/CRH pathway and the modulation of leptin signaling (14, 26).

Considering the available data on UCN3 and its family members, we hypothesized that UCN3 expression is increased in adipose tissue and blood in obese people and decreased with diabetes and thus affecting the cross talk between the brain and peripheral organs. Hence, this study investigated the following: (1) UCN3 levels in the blood and subcutaneous adipose tissue (SAT) from normal-weight and overweight individuals with and without T2D; (2) the association between UCN3 levels and physical, clinical, and biochemical markers; and (3) the effect of a 3-month physical exercise program on UCN3 levels in obesity and T2D.

## Methods

### Study Population

The study included 3 groups of adults: normal-weight without T2D (*n* = 37), overweight without T2D (*n* = 107), and overweight with T2D (*n* = 98). Written informed consent was obtained from all participants prior to starting this study, which was approved by the Review Board of Dasman Diabetes Institute and was conducted in line with the principles of the Declaration of Helsinki. People who performed regular physical exercise within the last 6 months prior enrollment to the study or those with history of major illness or medications not related to diabetes were excluded.

### Exercise Protocol and Anthropometric Measurements

All eligible subjects (*n* = 39 for each overweight group) were enrolled in a supervised exercise program at the Medical Fitness Center (MFC) of Dasman Diabetes Institute. Prior to exercise prescription each subject underwent the Cardiopulmonary exercise test “CPET” (COSMED, Quark, Italy) using electromagnetically braked cycle ergometer to measure the maximum heart rate (max HR) and maximum oxygen consumption (VO_2max_). Physical fitness was assessed thereafter to determine the muscle strength, endurance, and flexibility by performing grip strength (dynamometer), push-ups upper body strength, sit ups and forward bending test (upper and lower body flexibility). The exercise program involved a combination of both resistance training with treadmill or cycling and moderate intensity aerobic exercise. Each exercise session included 10 min warming up and cooling down steps at 50–60% of max HR with 40 min of the mentioned program at 65–80% of max HR. Under the supervision of qualified fitness professionals at MFC, subjects exercised 3 times per week for a period of 3 months. They were monitored to maintain the recommended heart rates during the training. Exercise effectiveness was assessed at the end of the 3-month exercise program using similar fitness tests performed at baseline.

### Blood and Tissue Sampling and Blood Analysis

SAT biopsies and venous peripheral blood were obtained at baseline and after the 3-month exercise and processed as previously reported (27). Briefly, Plasma and serum were prepared using vacutainer tubes and then aliquoted and stored at −80°C. SAT biopsies (200–500 mg) were obtained from the periumbilical area by surgical biopsy after a local anesthesia. Once removed, the biopsy was rinsed in cold PBS, divided into four pieces, and stored appropriately until assayed. Blood clinical markers, plasma inflammatory and metabolic markers were measured as previously reported (27). Lipid and glucose levels were measured with a Siemens Dimension Chemistry Analyzer (Diamond Diagnostics, Holliston, MA, USA). Hemoglobin A1c (HbA1c) levels were determined with the Variant™ device (Bio-Rad, Hercules, CA, USA). Plasma levels of UCN3 (#LS-F12902, Lifespan Biosciences, USA), hsCRP (Biovendor, Asheville, NC, USA) and insulin (Mercodia AB, Uppsala, Sweden) were assayed using ELISA kits, in accordance with the manufacturers' instructions. Plasma samples were diluted 1:10 prior to UCN3 measurement to fit within the detection range.

### Immunofluorescence Confocal Microscopy

Formalin-fixed, paraffin-embedded SAT sections (8 μm) from normal-weight and overweight with and without T2D subjects (*n* = 6 for each group) were deparaffinized and rehydrated followed by the antigen retrieval using DAKO solution at pH 6 (Dako, Glostrup, Denmark) in a pressurized cooker. The endogenous peroxidase was quenched using 3% H_2_O_2_ for 1 h at room temperature (RT). Sections were blocked with 5% fat-free milk for 1 h at RT, followed by 1% BSA for 1 h. Then the tissue sections were incubated at 4°C for overnight with primary antibodies (dilution 1:100). Primary antibodies used were anti-UCN3 antibody (ab79121, Abcam, UK) and anti-adiponectin (#5901, BioVision, USA). Antibody performance was assessed using negative controls (slides incubated without primary antibody or with unspecific IgG). Staining with Alexa Fluor 488-conjugated rabbit secondary antibody (A-11008, Invitrogen, USA) was used at 1:1,000 dilution and incubated at RT for 1 h. Human pancreas biopsies were used as positive controls for UCN3 expression. DAPI was used at 0.05% for nuclear staining. The sections were analyzed using a Zeiss LSM 710 confocal laser-scanning microscope (Zeiss, Germany), and 12 fluorescent images of the representative areas of the SAT were photographed at 40 × objective for each subject. The staining intensities were quantified using the ZEN software (Zeiss, Germany).

### Cell Culture and Treatments

Mouse preadipocyte (3T3-L1) and macrophage cells (RAW264.7) were obtained from ATCC (VA, USA), cultured and differentiated in their growth media as previously reported (28). For Indirect co-culture of 3T3L1 and RAW264.7 cells, media from RAW264.7cells were collected and sterile filtered (0.2 μm). The 3T3L1 cells were induced for differentiation using macrophage-conditioned media (MaCM) supplemented with differentiation cocktail [1% PS, 10% BCS, 0.5 mM 3-isobutyl1-methylxanthine (IBMX), 1 μM dexamethasone, and 1 μg/mL insulin] for 2 days. The medium was then changed to MaCM with 10% BCS and insulin (1 μg/mL) and replaced every 2 days up to day 8.

### Oil Red O Staining

After treatment with RAW264.7 cells conditioned media, 3T3-L1 cells were washed twice with PBS, fixed with 10% formalin for 1 h at RT, washed twice again with PBS, and incubated with 60% isopropanol for 5 min. Finally, cells were incubated with 0.3% Oil Red O solution for 20 min. Coverslips were washed with distilled water five times and mounted onto a slide. Images were captured using a panoramic digital slide scanner. Non-treated cells were used as control.

### Quantitative Real-Time PCR

Total RNA was extracted from SAT using RNeasy Lipid Tissue MiniKit (Qiagen, CA, USA). For cell lines, total RNA was extracted using TRIZOL reagent. cDNA was synthesized from total RNA samples using High Capacity cDNA Reverse Transcription Kit (Applied Biosystems, CA, USA). Conventional qRT-PCR was performed using the Applied Biosystem7500 system with Taqman gene expression assays normalized to *gapdh*. The primers used are presented in Table S1. The differences in gene expression between groups were assessed using the ΔΔCT method.

### Western Blot Analysis

Western blots were performed using 3T3-L1 cells. Whole cell extracts were prepared in RIPA buffer (50 mM Tris-HCl, pH 7.5, 150 mM NaCl, 1% Triton X-100, 1 mM EDTA, 0.5% sodium deoxycholate, and 0.1% SDS). Protein concentration was determined by the Bradford method using β-globulin as a standard. Twenty microgram of protein was prepared in sample loading buffer containing β-mercaptoethanol heated at 95°C for 10 min, then resolved on 12% SDS-PAGE gels. Proteins were then transferred at 100 V for 75 min onto PVDF membranes, blocked with 5% non-fat dried milk in Tris-buffered saline containing 0.05% Tween 20 (TBST) for 2 h at RT, and then probed with the anti-UCN3 primary antibody (1:1,000 dilution) (bs-2786R, Bioss Antibodies Inc., MA, USA) for overnight at 4°C. After washing, the membranes were incubated with Rabbit horseradish peroxidase conjugated secondary antibody (1:10,000 dilution) for 2 h at RT, and finally detection was performed using super sensitivity West Femto ECL reagent (Thermo Scientific, USA). Protein bands were visualized by chemiluminescence and the images captured by using the Versadoc 5000 system (Bio-Rad, USA). GAPDH was used as internal control to monitor for protein loading, with the anti-GAPDH (ab2302, Millipore, Temecula, CA). For densitometric analysis, the intensity of bands was determined using Quantity One Software (Bio-Rad, USA).

### Statistical Analysis

SPSS software (v25.0; SPSS Inc., IL, USA) was used to perform the statistical analyses. All descriptive statistics in the study are reported as mean ± standard deviation. A Chi-square test was used for categorical variables and the Wilcoxon non-parametric *t*-test was used for skewed variables. To evaluate the effects of groups, we conducted two-way repeated measures ANOVA test with *post-hoc* Bonferroni on the whole population. A paired *t*-test was used to determine the significance of differences in means within each group before and after exercise. Correlations between variables were calculated using the Spearman's correlation coefficient. Multivariable linear regression analysis was performed to examine the predictive effect of each factor with odd ratios (ORs), and their 95% confidence intervals for associated factors were estimated. All *p* < 0.05 were considered statistically significant.

## Results

### Plasma UCN3 Levels Are Affected by Obesity and T2D

Table 1 shows the characteristics of the study participants. Briefly, the overweight with T2D group showed more dysregulated lipid profile as reflected by lower HDL (*p* < 0.01) and higher TGL levels (*p* < 0.001) than the normal weight and overweight without T2D groups. Moreover, the overweight without T2D group displayed significantly higher systolic blood pressure (SBP) and lower maximal oxygen consumption (VO_2max_) levels than the normal-weight group (*p* < 0.01), whereas the overweight with T2D group had the highest SBP (*p* < 0.05) and lowest VO_2max_ (*p* < 0.01). Altered hormone profiles were also observed in the overweight groups, with higher levels of leptin, glucagon, glucagon-like peptide-1 (GLP-1), and gastric inhibitory polypeptide (GIP) than the normal-weight group (*p* < 0.05). The levels of circulating UCN3 were significantly decreased with increased body mass index (BMI) (*p* < 0.001) and significantly increased with T2D in the overweight group (*p* < 0.01) (Table 1).

**Table 1:** Physical, clinical, and biochemical characteristics of the subjects at baseline.

	**Normal-weight (*n* = 37)**	**Overweight non-diabetic (*n* = 107)**	**Overweight diabetic (*n* = 98)**	***Sig*.**
**PHYSICAL AND CLINICAL CHARACTERISTICS**				
Gender (M/F)	14/23	42/65	53/45	0.379
Age (years)	40 ± 11	42 ± 12	52 ± 9^$$$^	< 0.0001
BMI (kg/m^2^)	22.3 ± 1.9	31.9 ± 4.5^***^	32.3 ± 3.7	< 0.0001
PBF (%)	29.5 ± 5.7	36.6 ± 5.9^***^	36.1 ± 5.4	< 0.0001
Waist (cm)	77.1 ± 9.6	99.7 ± 12.2^***^	105.7 ± 10.56^$$^	< 0.0001
Hip (cm)	98.0 ± 5.6	113.7 ± 11.9^***^	111.4 ± 11.6	< 0.0001
Resting HR (beats/min)	80.9 ± 7.6	78.4 ± 10.4	82.3 ± 13.2	0.090
SBP (mmHg)	106.4 ± 10.1	115.6 ± 10.6^**^	119.1 ± 11.7^$^	< 0.0001
DBP (mmHg)	71.4 ± 6.6	74.3 ± 7.3	75.6 ± 6.3	0.091
WBC10	6.2 ± 1.6	6.4 ± 1.6	7.5 ± 2.0^$$$^	< 0.0001
VO_2_, max (ml/kg/min)	23.0 ± 5.1	18.0 ± 3.8^***^	15.7 ± 4.2^$$^	< 0.0001
**METABOLIC MARKERS**				
Cholesterol (mmol/l)	5.06 ± 1.07	5.10 ± 0.91	4.84 ± 1.33	0.248
HDL (mmol/l)	1.46 ± 0.43	1.31 ± 0.38	1.14 ± 0.37^$$^	0.0003
LDL (mmol/l)	3.11 ± 0.88	3.27 ± 0.85	2.97 ± 1.17	0.102
TGL (mmol/l)	0.89 ± 0.78	1.15 ± 0.63	1.64 ± 1.16^$$$^	< 0.0001
FPG (mmol/l)	5.12 ± 0.66	5.34 ± 0.85	8.37 ± 3.12^$$$^	< 0.0001
HbA1c (%) (mmol/l)	5.5 ± 0.4	5.7 ± 0.9	7.8 ± 1.8^$$$^	< 0.0001
Insulin (ng/ml)	2.74 ± 1.15	3.68 ± 2.19^*^	3.98 ± 1.95	0.012
	(37)	(39)	(62)	
C-peptide (ng/ml)	3.35 ± 4.43	5.77 ± 6.77	4.10 ± 5.50	0.073
HOMA-IR	0.60 ± 0.24	0.89 ± 0.37^*^	−−	< 0.0001
**HORMONAL MARKERS**				
Ghrelin (pg/ml)	920 ± 636	564 ± 225^***^	550 ± 232	< 0.0001
GIP (pg/ml)	431 ± 267	770 ± 684^*^	888 ± 585	0.005
GLP-1 (pg/ml)	255 ± 45	272 ± 50	295 ± 64^$^	0.004
Glucagon (pg/ml)	145 ± 46	159 ± 45	179 ± 55^$^	0.006
Leptin (ng/ml)	4.4 ± 2.6	9.2 ± 5.6^***^	7.9 ± 4.6	< 0.0001
Resistin (ng/ml)	3.0 ± 0.8	3.5 ± 1.7	3.3 ± 1.1	0.282
Visfatin (ng/ml)	3.6 ± 1.5	4.1 ± 3.1	4.5 ± 2.3	0.361
**INFLAMMATORY MARKERS**				
IL-1b (pg/ml)	10.2 ± 6.0	8.5 ± 3.0	8.5 ± 2.2	0.0560
IL-6 (pg/ml)	17.3 ± 4.8	17.1 ± 5.8	18.9 ± 8.7	0.2931
TNF-a (pg/ml)	138 ± 32	128 ± 38	140 ± 81	0.4590
RANTES (ng/ml)	7.5 ± 1.3	7.9 ± 1.6	8.2 ± 1.4	0.159
PAI-1 (ng/ml)	13.1 ± 5.2	15.2 ± 4.9	17.0 ± 6.4	0.007
hsCRP (μg/ml)	2.1 ± 1.2	5.2 ± 3.3	5.6 ± 4.7	0.174
**OTHER MARKERS**				
UCN3 (ng/ml)	11.99 (0.78–86.07)	6.27 (0.64–77.04)^***^	9.03 (0.77–104.92)^$$^	0.00015

*Data are presented as the mean ± SD. HR, heart rate; SBP, systolic blood pressure; DBP, diastolic blood pressure; VO_2_, max, maximum oxygen consumption; HDL, high-density lipoprotein; LDL, low-density lipoprotein; TGL, triglyceride; HbA1c, hemoglobin A1c; hsCRP, high-sensitivity CRP. Sig (significant) of 2-way ANOVA*.

* p < 0.05,

** p < 0.01,

*** p < 0.001 where * is significance between normal weight and overweight non-diabetic.

$ p < 0.05,

$$ p < 0.01,

$$$ *p < 0.001, where ^$^ is significance between overweight non-diabetic and diabetic*.

Considering the observed increased UCN3 levels with T2D, we further assessed UCN3 levels by dividing the T2D group into controlled and uncontrolled HbA1c groups (HbA1c < 6.5%; < 48 mmol/mol and HbA1c ≥ 6.5%; ≥ 48 mmol/mol, respectively). UCN3 levels were significantly higher in the uncontrolled HbA1c group than in the controlled HbA1c group (5.9 ± 3.6 and 11.7 ± 8.8 ng/ml, respectively; *p* < 0.05).

Spearman's correlation analysis between UCN3 plasma levels and various parameters was performed for all participants and separately for each group (Table 2). Briefly, UCN3 levels were positively correlated with TGL and visfatin/eNAMPT (*p* < 0.05) but were negatively correlated with insulin (*p* < 0.01) in the whole population and in the two overweight groups separately. Moreover, UCN3 levels were significantly correlated with FPG and HbA1c (*p* < 0.05) but were negatively correlated with RANTES (*p* < 0.05) in all subjects.

**Table 2:** Spearman Correlation ranking of UCN3 with subject characteristics at baseline.

	**All**	**All non-diabetic**	**Normal-weight**	**Overweight non-diabetic**	**Overweight diabetic**
Age (years)	0.07	−0.01	−0.25	0.09	0.11
BMI (kg/m^2^)	−0.02	−0.21^*^	−0.13	0.09	0.17
PBF (%)	−0.11	−0.17	−0.08	−0.16	−0.02
Waist (cm)	0.13	−0.03	0.01	0.04	0.12
Hip (cm)	−0.10	−0.10	−0.09	−0.04	−0.15
HR (beats/min)	−0.05	−0.10	0.27	−0.15	−0.06
SBP (mmHg)	0.17^*^	0.08	0.47	0.08	0.21^*^
DBP (mmHg)	0.11	0.04	0.30	0.04	0.13
VO_2max_ (ml/kg/min)	0.04	0.08	−0.09	0.06	0.21
Cholesterol (mmol/l)	0.09	0.09	−0.03	0.13	0.14
HDL (mmol/l)	−0.09	−0.04	0.04	−0.11	−0.01
LDL (mmol/l)	0.07	0.12	0.05	0.19	0.03
TGL (mmol/l)	0.21^**^	0.13	−0.11	0.24^*^	0.23^*^
FPG (mmol/l)	0.16^*^	0.05	−0.10	0.12	0.09
HbA1c (%)	0.20^**^	0.12	−0.08	0.21^*^	0.11
Insulin (ng/ml)	−0.22^**^	−0.28^**^	−0.18	−0.25^**^	−0.22^*^
C-peptide (ng/ml)	−0.07	−0.19^*^	−0.04	−0.14	0.17
HOMA1	−0.12	−0.23^*^	−0.17	−0.20	−0.16
IL-1b (pg/ml)	0.09	0.12	0.07	0.09	0.04
IL-6 (pg/ml)	0.12	0.04	0.03	0.09	0.11
TNF-a (pg/ml)	0.03	0.01	0.12	−0.11	0.08
RANTES (ng/ml)	−0.17^*^	−0.13	−0.39^*^	−0.02	−0.30^*^
hsCRP (μg/ml)	−0.10	−0.07	−0.30	−0.06	−0.15
Ghrelin (pg/ml)	0.13	0.11	−0.33	0.08	0.21
GIP (pg/ml)	−0.04	−0.12	−0.03	0.02	0.04
GLP-1 (pg/ml)	0.23^**^	0.17	0.19	0.27^*^	0.26^*^
Glucagon (pg/ml)	0.16^*^	0.14	0.35	0.19	0.16
Leptin (ng/ml)	−0.18^*^	−0.18	−0.11	−0.02	−0.21
PAI-1 (ng/ml)	−0.04	−0.09	0.11	−0.07	−0.04
Resistin (ng/ml)	−0.06	0.02	−0.15	0.12	−0.22
Visfatin (ng/ml)	0.36^**^	0.32^**^	0.26	0.37^**^	0.40^**^

Data are presented as the R-values. HR, heart rate; SBP, systolic blood pressure; DBP, diastolic blood pressure; VO_2max_, maximum oxygen consumption; HDL, high-density lipoprotein; LDL, low-density lipoprotein; TGL, triglyceride; HbA1c, hemoglobin A1c; hsCRP, high-sensitivity CRP. Significance of spearman correlation are as follows

* p < 0.05,

** *p < 0.01*.

To further assess the independent association of UCN3 with these markers, a multivariate stepwise linear regression analysis for UCN3 as a dependent variable was performed separately for the whole study population and separately for each group (Table 3 and Table S2). Independent associations were observed between UCN3 levels and FPG, HbA1c, GLP-1, and RANTES in the whole study population and in the overweight with T2D group (*p* < 0.05; Table 3). In the overweight without T2D group, only HbA1c was independently associated with circulating UCN3 levels (*p* < 0.05). Multivariate binary logistic regression analyses were then performed using either normal-weight or overweight without T2D as reference (Table 4 and Table S3). Plasma UCN3 levels were strongly related to adiposity for both overweight groups with and without T2D when compared to normal-weight (OR = 2.11 [1.84–4.11, 95% CI] and OR = 2.12 [1.59–3.10, 95% CI], *p* < 0.01, respectively).

**Table 3:** Multivariate Linear Regression analysis with UCN3 as dependent variable.

**Independent variables**	**All subjects**		**Overweight non-diabetic**		**Overweight diabetic**	
	**β coefficient**	***p* value**	**β coefficient**	***p* value**	**β coefficient**	***p* value**
Age	0.120	0.200	0.190	0.212	−0.071	0.606
Gender	−0.017	0.874	−0.294	0.067	−0.040	0.813
TGL	0.010	0.913	−0.015	0.909	0.045	0.721
FPG	−0.293	0.019^*^	−0.194	0.204	−0.380	0.042^*^
HbA1c	0.413	0.001^*^	0.501	0.001^*^	0.332	0.040^*^
Insulin	−0.125	0.166	−0.100	0.458	−0.268	0.078
GLP-1	0.310	0.010^*^	0.186	0.264	0.375	0.033^*^
Glucagon	−0.179	0.111	−0.009	0.949	−0.381	0.068
Leptin	−0.117	0.265	0.221	0.179	−0.238	0.170
Visfatin	0.091	0.325	0.242	0.078	0.198	0.174
RANTES	−0.298	0.001^*^	−0.162	0.200	−0.455	0.001^*^

* *p < 0.05 significant*.

**Table 4:** Odds Ratio analysis by binary logistic regression models compared to normal-weight subjects.

**Covariates**	**Normal weight**	**Overweight non-diabetic**		**Overweight diabetic**		**All overweight**	
	**Reference**	**OR (95% CI)**	***p* value**	**OR (95% CI)**	***p* value**	**OR (95% CI)**	***p* value**
UCN3	1.00	2.11 (1.84–4.11)	0.009^*^	2.12 (1.59–3.10)	0.008^*^	2.80 (1.94–4.13)	0.017^*^
TGL	1.00	1.86 (0.61–5.67)	0.278	0.83 (0.35–1.97)	0.677	2.86 (1.61–5.63)	0.037^*^
RANTES	1.00	1.00 (1.00–1.01)	0.937	1.00 (1.00–1.00)	0.068	1.00 (1.00–1.00)	0.486
C-Peptide	1.00	1.01 (1.00–1.02)	0.061	1.00 (0.99–1.00)	0.688	1.00 (1.00–1.02)	0.071
GLP1	1.00	1.01 (0.98–1.03)	0.441	1.03 (0.99–1.07)	0.058	0.99 (0.99–1.03)	0.174
Visfatin	1.00	1.00 (1.00–1.01)	0.219	1.00 (0.99–1.00)	0.437	1.00 (1.00–1.01)	0.384

Model adjusted for age and gender,

* *p < 0.05 significant*.

A pairwise comparison of UCN3 levels was also performed using various clinical and metabolic parameters in a subset of overweight subjects (BMI ≥ 30 kg/m^2^, *n* = 39 for each group) before and after exercise to assess the effect of 3 months of moderate physical exercise. We previously reported the impact of exercise on various markers and showed that it was more prominent in people without T2D, where adiposity markers [BMI, waist circumference, and percent body fat (PBF)] were significantly decreased, along with ameliorated cardiorespiratory markers (HR and VO_2max_) (25, 28). Table S4 summarizes the markers significantly affected by exercise in our cohort. Despite an increase in UCN3 plasma levels with exercise in the overweight without T2D and overweight with T2D groups, statistical significance (*p* < 0.05) was reached only in the overweight without T2D group.

### UCN3 Expression Is Increased in SAT From Overweight Individuals and Decreased by Physical Exercise and Inflammation

**Figure 1:**
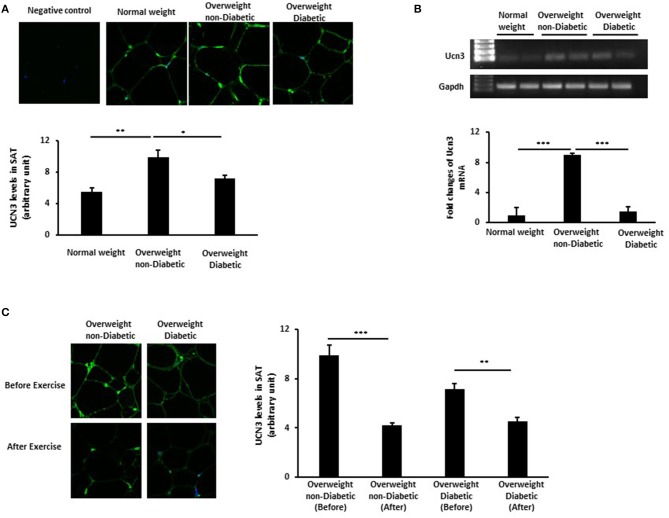
UCN3 expression levels in subcutaneous adipose tissue (SAT). **(A)** Representative confocal immunofluorescence images illustrating UCN3 expression and localization in SAT from normal-weight, overweight people with and without T2D (*n* = 10 for each group). Staining quantification of SAT was performed as detailed in Materials and Methods and data are presented as arbitrary unit. **(B)** mRNA expression of Ucn3 in the subcutaneous adipose tissue (SAT) of participant groups. mRNA levels were measured by quantitative real-time PCR using SAT from normal-weight and overweight people with and without T2D and PCR products were run on agarose gel. GAPDH was used as an internal control for normalization and data are presented as fold changes compared with the findings in the normal-weight participant. **(C)** Representative confocal immunofluorescence images illustrating UCN3 expression and localization in SAT from overweight individuals with and without T2D before and after 3-month physical exercise (*n* = 6 for each group). Staining quantification of SAT was performed as detailed in Materials and Methods and data are presented as arbitrary unit. The *p*-value was determined using the Wilcoxon test was used for comparisons between each 2 groups separately and a paired *t*-test for intragroup comparisons before and after exercise. *p < 0.05, **p < 0.01, ***p < 0.001.

To elucidate the role of UCN3 in obesity and T2D, we assessed UCN3 expression levels in SAT from representative individuals from the three groups. Our results revealed an opposite pattern of UCN3 expression in SAT from those seen in blood (Figures 1A,B). UCN3 expression was significantly increased at both the protein (*p* < 0.01) (as observed by confocal IF staining) and mRNA (*p* < 0.001) levels in the overweight without T2D individuals compared with the normal-weight individuals. However, UCN3 expression was significantly decreased in the overweight with T2D individuals compared with those without T2D (*p* < 0.05 and *p* < 0.001 for protein and mRNA, respectively). As a control, adiponectin staining was assessed in SAT from the same individuals and revealed lower levels in the overweight without T2D individual compared with the normal-weight individual and was further decreased in the overweight with T2D individual (Figure S1). We also examined the effect of a 3-month moderate physical exercise program on UCN3 expression levels in SAT from overweight individuals. SAT UCN3 expression levels were significantly reduced after exercise in both overweight groups regardless of the presence or absence of T2D (*p* < 0.01), as indicated by the clear decrease in UCN3 protein staining assessed by IF confocal microscopy (Figure 1C).

**Figure 2:**
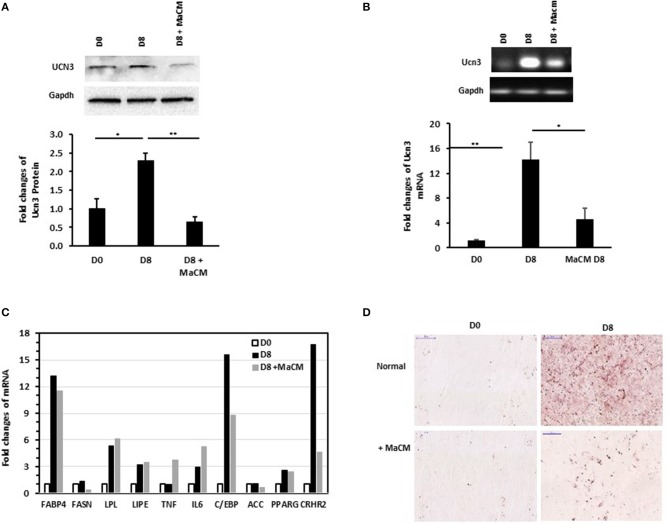
Expression levels of Ucn3 before and after differentiation of 3T3 preadipocytes. Protein **(A)** and mRNA **(B,C)** levels were measured by Western blot and quantitative real-time PCR, respectively, using 3T3-L1 preadipocytes (D0), adipocytes differentiated for 8 days (D8) with and without macrophage conditioned media (MaCM). Data are presented as fold changes in differentiated adipocytes compared with the findings in preadipocytes from 3 independent experiments**. (D)** Representative images for Oil Red O staining illustrating differentiation under normal and MaCM conditions. Images were taken at magnification of 40X. *p < 0.05 and **p < 0.01.

We previously reported the differential patterns of stress and inflammation markers in the SAT of normal-weight and obese individuals, as well as between individuals with and without T2D (25, 28). This, along with the correlation of UCN3 with RANTES in our current study, prompted us to assess the effect of inflammation on its expression levels during preadipocyte differentiation with and without macrophage conditioned media (MaCM). By using the murine 3T3-L1 cells, we showed that both UCN3 mRNA and protein were expressed in both the preadipocyte (day 0) and adipocyte (day 8) phenotypes (Figures 2A,B). Furthermore, adipogenic stimuli–induced differentiation markedly increased both UCN3 mRNA and protein levels in mature adipocytes (day 8, *p* < 0.05) (Figures 2A,B) as well as UCN3 receptor (CRHR2) (Figure 2C). Finally, the differentiation of 3T3-L1 preadipocytes in the presence of MaCM strongly reduced UCN3 levels in differentiated adipocytes at both mRNA and protein levels (*p* < 0.05) and its receptor, as well as lipid droplet accumulation (Figures 2C,D). The increased levels of expression of genes related to lipogenesis (FASBP4, FASN), lipolysis (LPL, LIPE), inflammation (IL6) as well as C/EBP and PPARG indicated that cell differentiation is achieved both in the presence and absence of MaCM (Figure 2C).

## Discussion

This study is the first to report UCN3 levels in blood and SAT from normal and overweight adults with and without T2D and the modulation of UCN3 levels by physical exercise. The main findings of this study are: (1) plasma UCN3 levels decreased with BMI; (2) UCN3 plasma levels were independently associated with glycemic index (FPG, HbA1c) and were significantly different between overweight individuals with and without T2D; (3) the UCN3 expression in SAT was increased with BMI and blunted with T2D; and (4) a 3-month supervised exercise protocol showed a significant reduction of UCN3 expression in both overweight group SAT.

Our findings of impaired UCN3 levels in obesity and T2D with opposite pattern between plasma and SAT indicate the complexity of mechanisms under these conditions. Despite the well-established role of impaired feedback in the inhibition of insulin secretion in hyperinsulinemia and insulin resistance (29, 30), multiple central and peripheral endocrine and inflammatory pathways are also disturbed. UCN3 was reported to be co-released with insulin from β-cells and acts in a paracrine fashion on delta cells to trigger the release of somatostatin and limit further insulin release (12). However, the role of UCN3 in obesity and diabetes is not well-understood. To date, most studies into urocortins have focused on UCN1 and UCN2 in various diseases including heart failure (HF) and have revealed compelling data on the endogenous compensatory role of UCN1 and UCN2 and their cardioprotective effects (31, 32). During gestation, the placenta does not secrete significant UCN2 and UCN3 into the plasma despite the increased plasma CRF levels (33). Placental UCN2 and UCN3 levels likely function as autocrine/paracrine messengers during pregnancy. Interestingly, initial studies have suggested that a negative relationship exists between UCNs concentration and their pharmacokinetics (34). Furthermore, UCN3 is present in urine, and levels are significantly increased in patients with obstructive sleep apnea (35). These findings reflect variations in UCN patterns, particularly UCN3, depending on the tissue and disease.

In line with this and contrary to the pattern of circulating UCN3 observed in our study population, we found that UCN3 expression SAT increased with obesity and blunted with T2D in this tissue likewise previous reports showing decreased levels of UCN3 in β-cells people with diabetes (12). In plasma, our data demonstrate an association between UCN3 levels and glycemic index in individuals with and without T2D, thus further supporting the involvement of UCN3 in glucose homeostasis and/or insulin regulation. Within the overweight group, the significantly high OR (2.24) of circulating UCN3 in individuals with T2D compared with those without T2D supports the involvement of UCN3 in diabetes (Table S3). The strong correlation observed between UCN3, TGL, and visfatin in plasma further indicates global metabolic disturbance due to obesity and hyperglycemia in our study population. However, the role and involvement of circulating UCN3 in diabetes are yet to be elucidated.

Although UCN3 is expressed in human adipocytes and adipose tissue (36, 37), its role in obesity and diabetes has not been reported. Giamouridis et al. (38) demonstrated that UCN3 gene transfer showed promising potential as a treatment for HF and had significant beneficial effects on glucose control, weight loss, and liver fat reduction. UCN3 was also reported to circulate in normal human plasma, and its possible sources included a range of tissues such as heart, brain, kidney, stomach, intestine, pancreas, muscle, and vasculature, thus suggesting that UCN3 has a hormonal role (3, 39). However, the source, concentrations, and forms of UCN3 in plasma remain to be determined. Similarly, the contribution and amount of UCN3 secreted from adipose tissue into the circulation remain to be elucidated, and it is possible that adipose tissue UCN3 plays a paracrine/autocrine hormonal role. Nevertheless, the opposite pattern of UCN3 observed in the SAT and plasma in the present study does not support a significant contribution or secretion from SAT to circulating levels of UCN3. Our findings do not rule out the possibility that UCN3 may be released from adipose tissue to a greater extent into the plasma under other conditions and diseases, in particular from visceral adipose tissue where UCN3 might be more abundant (32). Nevertheless, we can exclude the significant uptake of UCN3 by SAT owing to diabetes because of the observation of opposite UCN3 profiles in plasma and SAT and the fact that UCN3 mRNA levels paralleled those of protein levels in SAT.

On the other hand, the observation that the expression of UCN3 mRNA and protein in SAT was increased by the adiposity but not by the concomitant presence of T2D in overweight subjects might indicates that, UCN3 expression is primarily associated with SAT expansion. In addition, in diabetic obese SAT, we and others have previously reported further increased inflammation and ER stress (24, 40). This suggests that an altered local microenvironment may affect UCN3 expression in obese diabetic adipose tissue. Indeed, our experiments using conditioned media from macrophages to culture 3T3-L1 adipocytes markedly repressed UCN3 gene expression (Figure 2). Moreover, association between RANTES as inflammatory mediator and UCN3 was found in the obese subjects with diabetes but not without diabetes (Table 2). Thus, it appears that UCN3 up-regulation in human SAT is closely related to the metabolic derangements associated with greater adiposity (hypertrophy). UCN3 might participates in local adaptive inflammation and stress for maintaining tissue homeostasis but this effect might be blunted with excessive and chronic cellular stress, as in diabetes, and reflected by decreased levels of UCN3 in the SAT. Similar pattern have been observed in our previous work on the heat shock protein 60 (HSP60) were attenuated in normal-weight subjects with diabetes, in comparison with non-diabetic controls, along with increased expression of the inflammatory markers IL-6 and TNF-α, as observed in our overweight subjects (25).This suggests an autocrine/paracrine role of UCN3 in SAT which is further supported by the opposite trend observed in plasma. Interestingly, The Odd Ratios (OR) analysis for plasma UCN3 showed that UCN3 levels are more affected by the obesity than diabetes (see Table 4).

Furthermore, SAT expansion in obesity is known to create hypoxic microenvironment which amplifies inflammation and hyperglycemia (41). Interestingly and more recently, it was shown that in WAT hypoxia, lipolysis was significantly increased by activating Ucn2/3–CRHR2–cAMP–PKA pathway in an autocrine/paracrine manner (42). In line with this, transgenic UCN3+ mice exhibited a favorable metabolic phenotype resisting obesity and hyperglycemia with improved fatty acid metabolism. This supports that UCN3 plays a role in maintaining energy and glucose homeostasis in adipose tissue (9). Finally, stem cells differentiation in the SAT of diabetic individuals is impaired (43). T2D and aging are also associated with increased SAT stem cell senescence, reduced adipogenesis and hypertrophic expansion (44). It is worth to mention here, that our diabetic subjects are more aged than the non-diabetic ones. Altogether, those processes might explain the impaired UCN3 expression in diabetic compared to non-diabetic subjects.

Physical exercise reduces the risk of chronic metabolic diseases by modulating inflammatory and stress responses. Our data showed that a 3-month supervised exercise program significantly increased circulating UCN3 levels in the overweight without T2D group concomitant with the decreased PBF and waist circumference and improved VO_2max_. The increase in UCN3 levels was also consistent with reduced insulin levels. We and others have previously reported the beneficial effects of regular exercise in individuals without T2D compared with those with T2D (25, 28, 45). Our findings demonstrate that the exercise-mediated increase in circulating UCN3 was not translated to an increase in SAT UCN3. On the contrary, SAT UCN3 levels were decreased in both the overweight groups. A lack of parallel changes between SAT and circulating UCN3 levels raises questions about the role of UCN3 from different tissue sources and its mode of action but partly supports the suggested autocrine/paracrine role of UCN3 in SAT. Interestingly, the SAT UCN3 expression pattern between the three groups was similar to the one observed in PBMCs (data not shown). The difference in the patterns of circulating and SAT UCN3 indicates that dysregulation is not limited to SAT; therefore, it would be of interest to investigate its status in other UCN3-producing tissues. Data on the effect of exercise on UCN3 in health and diseases are limited. However, previous studies suggested a link between exercise and the central responsiveness of the hypothalamic-pituitary-adrenal axis, including the activation of the CRF system and increased corticosterone level secretion (46, 47).

Despite our novel findings, this study has some limitations. First, we did not have access to visceral adipose tissue biopsies, which would be more relevant to the pathophysiology of diabetes and obesity. Second, urine samples were not collected to assess UCN3 clearance in the various phenotypes. Third, despite our adjustment for many confounders, we cannot exclude that some of the associations we have observed can be due to other variables including; the lack of detailed medication of our subjects and controlled dietary intake which may have affected UCN3 levels and efficacy of physical exercise despite the marginal observed body-weight loss. Furthermore, diabetes is associated with aging, and it was difficult to find age-matched healthy obese controls. Finally, we did not consider the potential contribution of UCN3 gene polymorphism in our data analysis as we did not have to this information. UCN3 gene polymorphisms have been reported to contribute to adiposity and intramuscular adipose deposition (18). However, the strengths of this study include that it involved high-risk group of obese adults with or without T2D who underwent a supervised moderate exercise protocol as a behavioral approach to improve global health without diet restriction. However, further studies are required to understand the cross talk between UCN3 levels and insulin resistance, and its role in adipose tissue, including the status of CRF receptors in SAT. These studies will help elucidate the mechanisms involved in the differential patterns seen between circulating and endogenous UCN3 levels.

## Conclusions

We found that UCN3 is associated with markers of glucose metabolism in humans and that there is differential dysregulation of UCN3 levels with obesity and T2D in plasma and SAT. Our results also show that moderate physical exercise differentially modulates UCN3 levels in the body and provides further evidence for the effect of physical exercise in mitigating metabolic stress linked with obesity and insulin resistance.

## Data Availability Statement

All generated data and resources are reported in this manuscript and there is no other data to be provided.

## Ethics Statement

Written informed consent was obtained from all participants prior to starting this study (RA-2010-003), which was approved by the Review Board of Dasman Diabetes Institute and was conducted in line with the principles of the Declaration of Helsinki.

## Author Contributions

SK, AK, and AT designed the study, analyzed the data and wrote and revised the manuscript. SK performed the experiments. SD performed statistical analysis. JT contributed to the discussion and the revision of the manuscript. DM, MH, JA, and FA-M revised the manuscript.

### Conflict of Interest

The authors declare that the research was conducted in the absence of any commercial or financial relationships that could be construed as a potential conflict of interest.

## Abbreviations

DBP: diastolic blood pressure
HbA1c: hemoglobin A1c
HDL: high-density lipoprotein
HR: heart rate
hsCRP: high-sensitivity CRP
LDL: low-density lipoprotein
PBF: percent body fat
SAT: subcutaneous adipose tissue
PBMC: peripheral blood mononuclear cell
SBP: systolic blood pressure
TGL: triglyceride
UCN: Urocortin VO_2,max_, maximum oxygen consumption
WC: waist circumference.
