# Supplementary material for: Urocortin 3 Levels Are Impaired in Overweight Humans With and Without Type 2 Diabetes and Modulated by Exercise
Source: Front Endocrinol (Lausanne). 2019 Nov 6;10:762. doi: 10.3389/fendo.2019.00762 (PMC6851015; doi:10.3389/fendo.2019.00762)
Supplement: Supplementary file 1 [file Data_Sheet_1.pdf]

Urocortin 3 levels are impaired in overweight humans with and without type 2 diabetes and modulated by exercise

Sina Kavalakatt[1#], Abdelkrim Khadir[1#], Dhanya Madhu[1], Maha Hammad[1], Sriraman Devarajan[2], Jehad Abubaker[1], Fahd Al-Mulla[2], Jaakko Tuomilehto[2,3] and Ali Tiss[1*]

[1] Biochemistry and Molecular Biology department, Research Division, Dasman Diabetes Institute, Kuwait
[2] Research Division, Dasman Diabetes Institute, Kuwait
[3] Department of Public Health, University of Helsinki, Helsinki, Finland; Department of Public Health Solutions, National Institute for Health and Welfare, Helsinki, Finland

Supplementary Information

Table S1: List of primers used for RT-PCR analysis

Gene	***Catalogue # or sequence***
Human Ucn3	Hs00846499 *(Applied Biosystem, USA)*
Human Gapdh	Hs02786624 *(Applied Biosystem, USA)*
Mouse Ucn3	Mm00453206 *(Applied Biosystem, USA)*
Mouse Gapdh	Mm99999915 *(Applied Biosystem, USA)*
PPARG	Mm0*0440940 (Applied Biosystem, USA)*
CRHR2	Mm0*0438308 (Applied Biosystem, USA)*
FABP4	Mm0*0445878 (Applied Biosystem, USA)*
FASN	Mm00*662319 (Applied Biosystem, USA)*
LPL	Mm00*434764 (Applied Biosystem, USA)*
LIPE	Mm00*495359 (Applied Biosystem, USA)*
TNF	Mm00*443258 (Applied Biosystem, USA)*
IL6	Mm00*446190 (Applied Biosystem, USA)*
C/EBP	*5'-GAACAGCAACGAGTACCGGGTA-3'*<br>*5'-GCCATGGCCTTGACCAAGGAG-3'*
ACC	*5'-GAACAGCAACGAGTACCGGGTA-3'*<br>*5'-GCCATGGCCTTGACCAAGGAG-3'*

Table S2: Multivariate Linear Regression analysis with UCN3 as dependent variable

**Independent variables**	**Normal-weight**		**All Non-Diabetic**	
	β coefficient	p value	β coefficient	p value
Age	-0.374	0.929	-0.022	0.840
Gender	0.389	0.429	-0.033	0.883
TGL	-0.057	0.511	-0.090	0.828
FPG	0.423	0.922	-0.080	0.498
HbA1c	-0.184	0.512	0.365	0.588
Insulin	-0.062	0.766	**-0.045**	**0.007***
GLP-1	-0.128	0.904	0.140	0.731
Glucagon	0.117	0.889	-0.088	0.403
Leptin	0.075	0.854	-0.055	0.545
Visfatin	0.355	0.903	0.221	0.717
RANTES	-0.143	0.561	-0.217	0.099

Table S3: Odds Ratio analysis by binary logistic regression models

Covariates	Overweight Non-Diabetic	Overweight - Diabetic	
	**Reference**	**OR (95% CI)**	**p value**
**UCN3**	1.00	2.24 (1.01 – 4.96)	**0.019***
**TGL**	1.00	1.77 (1.13 – 2.79)	**0.014***
**RANTES**	1.00	1.12 (1.01 – 1.27)	**0.031***
**C-Peptide**	1.00	1.03 (0.99 – 1.08)	0.072
**GLP1**	1.00	1.01 (1.00 – 1.05)	**0.039***
**Visfatin**	1.00	1.04 (1.00 – 1.11)	**0.030***

Model adjusted for age and gender, ** p<0.05 significant*

Table S4: Effect of exercise in overweight subjects

		**Non-Diabetic (n=39)**	**Diabetic (n=39)**
Gender (M/F)		17/22	25/14
BMI (kg/m2)	Before	31.04±4.12	32.17±3.41
	After	**30.28±4.41****	31.86±3.26
PBF (%)	Before	35.84±5.78	35.45±5.34
	After	**34.81±6.48****	**34.80±5.35**$^{\$}$
Waist (cm)	Before	98.25±8.35	107.74±8.76
	After	**94.61±8.29****	**105.34±9.12**$^{\$}$
VO2, max (ml/kg/min)	Before	17.95±3.68	17.69±3.59
	After	**21.22±4.72*****	18.52±4.42
Cholesterol (mmol/l)	Before	5.21±0.97	4.78±1.27
	After	5.19±1.03	**4.29±0.91**$^{\$}$
GLU (mmol/l)	Before	5.21±0.62	8.18±2.98
	After	**5.51±1.12***	7.89±2.74
HbA1c (%)	Before	5.93±1.21	7.71±1.95
	After	5.70±0.48	**7.10±1.38**$^{\$\$}$
Insulin (ng/ml)	Before	4.18±1.57	2.96±1.22
	After	**3.41±1.28***	2.80±1.53
Ghrelin (ng/ml)	Before	647.63±205.29	617.41±228.0
	After	677.06±203.54	**671.90±272.3**$^{\$}$
GIP (ng/ml)	Before	807.51±809.51	911.26±535.3
	After	**437.04±284.1****	**605.28±358.4**$^{\$\$}$
UCN3 (ng/ml)	Before	6.68 (0.64 − 65.8)	9.49 (0.77 − 104.92)
	After	**8.37 (0.76−72.8)***	10.56 (1.06 − 98.3)

*Only statistically significant data are presented. Data are presented as the mean ± SD. VO_2 max (maximum oxygen consumption), and HbA1c (hemoglobin A1c). Sig (significant) of paired-t test. * p<0.05, **p<0.005, ***p<0.0005 where * is significance overweight non-diabetic before and after exercise. $^{\$}$ p<0.05, $^{\$\$}$ p<0.005, $^{\$\$\$}$p<0.0005, where $^{\$}$ is significance between overweight diabetic before and after exercise.*

Figures

Figure S1:

(A) Representative confocal immunofluorescence images illustrating UCN3 expression in positive and negative controls using pancreas biopsies.

(B) Representative confocal immunofluorescence images illustrating adiponectin abundance in SAT from normal-weight, overweight with and without people.
