# Supplementary material for: Urocortin 3 Levels Are Impaired in Overweight Humans With and Without Type 2 Diabetes and Modulated by Exercise
Source: Front Endocrinol (Lausanne). 2019 Nov 6;10:762. doi: 10.3389/fendo.2019.00762 (PMC6851015; doi:10.3389/fendo.2019.00762)
Supplement: Supplementary file 2 [file Presentation_1.pptx]

## Slide 1
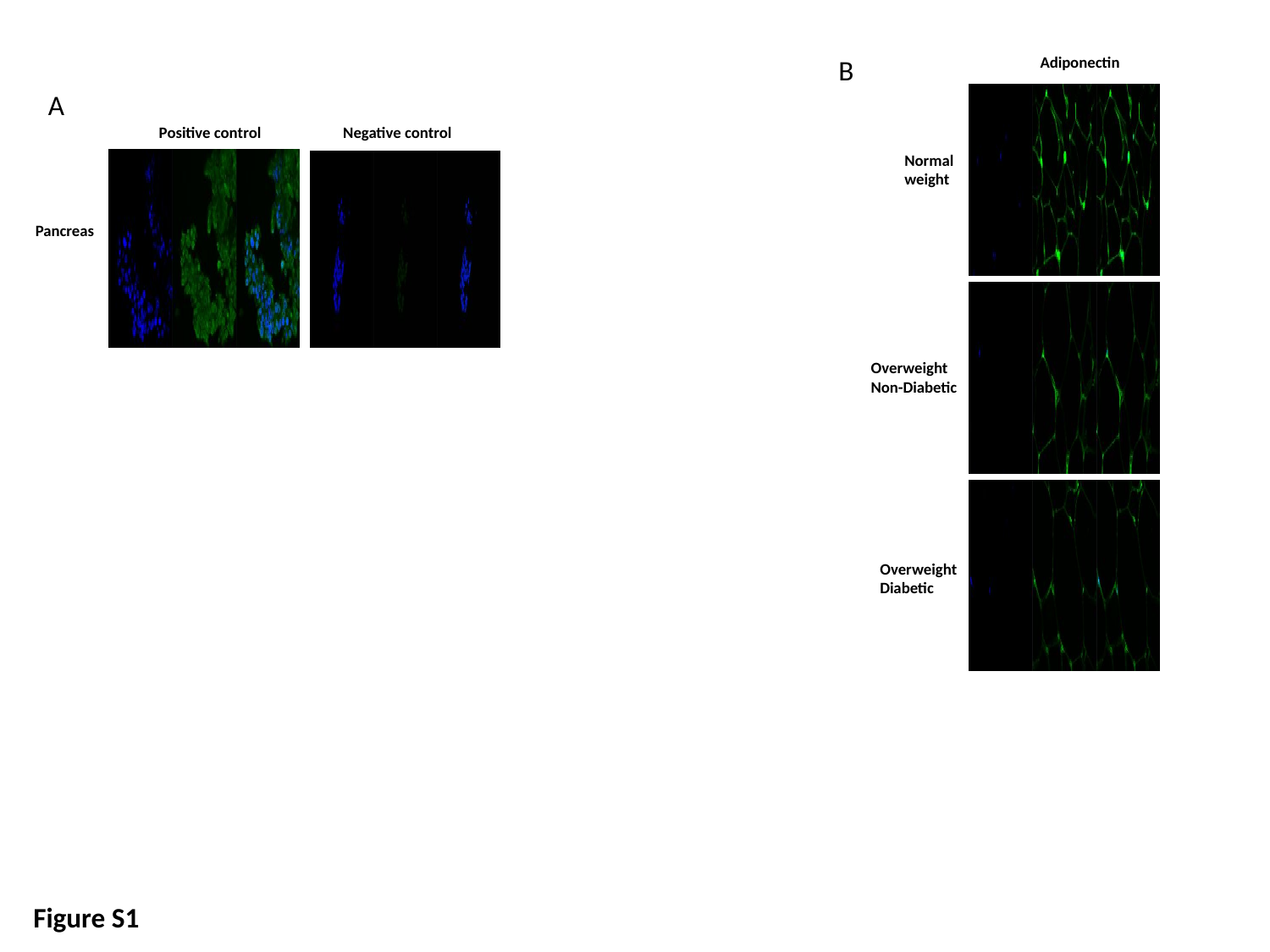

B
Normal
weight
Overweight
Non-Diabetic
Overweight
Diabetic
Adiponectin
A
Positive control
Negative control
Pancreas
Figure S1
